# Growth Differentiation Factor 15 Induces Gastric Fundus Contraction Involving Cholinergic Excitation: Morphofunctional Evidence in Rodent Models

**DOI:** 10.1111/jcmm.70629

**Published:** 2025-06-04

**Authors:** Rachele Garella, Francesco Palmieri, Livio Tarchi, Alessia Tani, Flaminia Chellini, Giulia Guarnieri, Annamaria Morelli, Caterina Bernacchioni, Paolo Rovero, Valdo Ricca, Giovanni Castellini, Chiara Sassoli, Roberta Squecco

**Affiliations:** ^1^ Department of Experimental and Clinical Medicine, Section of Physiological Sciences University of Florence Florence Italy; ^2^ Department of Health Sciences, Psychiatry Unit University of Florence Florence Italy; ^3^ Department of Experimental and Clinical Medicine, Section of Anatomy and Histology, Imaging Platform University of Florence Florence Italy; ^4^ Department of Experimental and Clinical Biomedical Sciences “Mario Serio” University of Florence Florence Italy; ^5^ Interdepartmental Research Unit of Peptide and Protein Chemistry and Biology (Peptlab), Department of Neurosciences, Psychology, Drug Research and Child Health (NeuroFarBa) University of Florence Florence Italy

**Keywords:** GFRAL, macrophage inhibitory cytokine 1, nausea, RET, satiety, signalling peptides, smooth muscle

## Abstract

Growth Differentiation Factor 15 (GDF15) is a peptide from the transforming growth factor (TGF)‐β superfamily, typically found at low levels in mammalian tissues, but significantly upregulated during cellular stress or injury. Initially recognised for its role in inducing anorexia and vomiting, GDF15 is now seen as a broader regulator of homeostasis, although its effects on gastrointestinal function remain unclear. This study examined GDF15's impact on the gastric fundus, a key region for appetite regulation. In ex vivo rodent gastric smooth muscle, GDF15 receptors were detected, and exposure to GDF15 caused smooth muscle depolarization, leading to increased mechanical activation. Morphological analyses revealed changes in the contractile apparatus, resembling cholinergic excitatory pathways. These effects were blocked by atropine, indicating muscarinic receptor involvement. Taken together, these findings suggest that GDF15 enhances gastric contractility by influencing cholinergic tone. Further studies will shed light on its mechanism of action and on the potential translational perspective of current results, elucidating whether elevated plasma levels of GDF15 observed in several physiological and pathological conditions can also have repercussions in gastric physiology, appetite regulation and weight loss.

## 1 Introduction

Growth differentiation factor 15 (GDF15) also known as macrophage inhibitory cytokine (MIC)‐1, is a peptide hormone belonging to the transforming growth factor (TGF) β protein superfamily [1, 2]. Its effects are primarily mediated by a peculiar signalling complex consisting of a receptor first identified in the brainstem [3], the Glial‐derived neurotrophic Receptor Alpha‐Like (GFRAL) and its co‐receptor Rearranged during Transfection (RET) tyrosine kinase receptor [4]. Nevertheless, the presence of GFRAL has since been identified in other brain areas as well as in peripheral tissues, such as liver, visceral fat tissue, kidney, gastrointestinal tissue, cardiac and skeletal muscle, and aortic smooth muscle [5]. However, to date, there is no evidence of GFRAL expression in the gastric wall, at least in non‐cancerous tissues.

In humans, GDF15 shows a pleiotropic action and is physiologically expressed at a low concentration in several tissues [6]. To the Author's knowledge, no definite data concerning GDF15 concentration specifically in the gastric fundus wall are available under normal conditions [7]. Serum GDF15 levels in humans typically range approximately from less than 200 to 1,200 pg/mL in healthy individuals [8, 9] with a physiological mean serum concentration of about 450 pg/mL [10]. However, plasma levels have been shown to significantly increase not only in malignant conditions (reaching up to 10,000–100,000 pg/mL) [7], but also in healthy subjects as a function of age (being 2,152 pg/mL at age ≥ 80 years) [11] or in pregnant women (19,311 pg/mL) [11]. Notably, the physiological mean serum concentration of GDF15 in mice was reported to be about 100 pg/mL [12].

First mainly described as a molecule capable of inducing anorexia and vomiting [1], GDF15 has since gained significant attention for its role in various physio‐pathological conditions where it is remarkably upregulated, such as in response to cell stress or tissue injury [13, 14, 15, 16]. Pregnancy, exercise, aging, organ failure, inflammatory disease, neoplasia, cancer cachexia and mitochondrial diseases are only some of the conditions where augmented circulating levels of GDF15, in the order of ng/mL, have been detected [6, 7, 17, 18, 19, 20]. Notably, GDF15 has been extensively studied in the context of metabolism regulation [19, 21], appetite [22], weight loss [15], and anorexia [1, 13, 23]. However, as previously mentioned, its impact on gastrointestinal function remains less understood. As a central component of the gastrointestinal system, the stomach plays a key role in digestion, primarily functioning as a reservoir for ingested food, facilitating the gradual breakdown of macronutrients, and promoting their movement into the intestine. In addition, the gastric fundus is, among other things, the site of afferent signals that, following distension, indicate gastric fullness and satiety, thus modulating food intake [24]. By contrast, increased excitation in the gastric fundus impairs accommodation and accelerates gastric emptying [25]. Therefore, two key aspects of stomach physiology are smooth muscle cell (SMC) excitability and tone, both of which are essential for maintaining proper stomach accommodation, internal pressure and gastric motility. In fact, SMC excitability enables the generation of action potentials which, in turn, allow for muscle contraction (i.e., excitation‐contraction coupling), thus determining an increase in gastric tone and peristaltic movements. SMC excitability is critical for triggering gastric muscle activity, as well as to guarantee proper muscular tone—protecting the organ from excessive internal pressure, ensuring the efficient breakdown of gastric content, and facilitating its movement into the duodenum. To date GDF15 has mainly been described as being inhibited at the gastric level during fasting and promoted by overfeeding, while under the influence of physiological and pathological factors, GDF15 secretion at the gastric level increases with age, is elevated in females compared to malesand is strongly upregulated by neoplasia [20, 26].

A better definition of GDF15's action on gastric tissue, however, seems warranted. In fact, although its receptor has been identified in different parts of the intestine [5], whether GRAL/RET receptors are expressed in the stomach has yet to be described [27] and GDF15's activity on gastric functionality remains unexplored, even if GDF15 has been suggested to modulate gastric emptying through vagus nerve [28]. A working hypothesis suggests that GDF15 may modulate food intake, increasing gastric tone to facilitate gastric emptying, in light of its role in promoting anorexia and vomiting [1]. This hypothesis, if proven empirically, could lead to widening the characterisation of GDF15 as a key homeostatic controller, not only at the central level, but also within peripheral tissues [27].

Therefore, the primary objective of this study was to analyse the effects of GDF15 on gastric muscle tissue samples of rodent models (i.e., mouse and rat), through a trans‐disciplinary approach, combining morphological and functional studies. Based on our previous know‐how [24], the current study explored the effect of GDF15 on the gastric fundus of the mouse. More specifically, we investigated SMC excitability along with contractile machinery organisation in the gastric fundus and proposed a potential pathway for GDF15's action in gastric fundus.

## 2 Materials and Methods

### 2.1 Animals, Gastric Fundus Muscle Strip Preparation and Treatments

Two different rodent models (i.e., mice and rats) were used for ex vivo experiments. Specifically, 12‐week‐old C57BL/6 female mice (*n* = 15, Charles River, Lecco, Italy) and Sprague–Dawley female rats (*n* = 12; Envigo, San Pietro al Natisone, Udine, Italy) were housed in the Laboratory Animal Facility in a temperature (23°C ± 1 °C) and humidity‐controlled room on a 12‐h light/dark cycle. They were fed a standard laboratory diet (Global diet; Mucedola srl, Settimo Milanese, Milan, Italy) and water *ad libitum*, up until sacrifice. To reduce animal suffering, mice were sacrificed by rapid cervical dislocation [24] and rats by beheading [29].

After sacrifice, the stomach was immediately extracted from the abdomen, the gastric fundus was rapidly dissected and opened along the smaller curvature following procedures previously reported [24]. The mucosa, submucosa and serous coat were accurately removed under a dissecting microscope in order to expose the smooth muscle layer. The smooth muscle layer was then cut to obtain 3–4 longitudinal tissue strips. The strips were then treated with: GDF15 (4 nM), or carbachol (CCh; 1 μM, 35 min), or atropine (1 μM, 30 min) [30, 31]. In particular, atropine was used alone or in combination with GDF15. In a set of experiments, atropine was also used in combination with CCh. The concentration of the chemicals is given as the final concentration in the bath solution. Untreated samples were used as control (CTRL).

The GDF15 concentration of 4 nM was chosen on the basis of previous literature [32, 33, 34, 35], in order to mimic GDF15 effects occurring in stress conditions. In other words, a final concentration was reached to resemble the range of plasma levels described in human patients with chronic illness or in pregnancy [1, 11], conditions often accompanied by appetite dysregulation and/or nausea/vomiting and eventually weight loss. This target dose (~100 ng/mL) approximately corresponded to 4 nM. To test the eventual efficacy of lower doses, concentrations in electrophysiological experiments were progressively halved to 2 and 1 nM. Higher doses were not tested in this study on rodents because they would not represent likely concentrations in vivo [36]. All the chemicals were purchased from Sigma‐Aldrich (St. Louis, MO, USA) except for GDF15 (PeproTech Inc., Rocky Hill, NJ, USA).

Electrophysiological records were performed at different times of exposure, namely for 5, 20, 35 and 50 min in the presence of GDF15 in the bath chamber. These relatively short periods of observation were chosen given the structural resemblance of GDF15 to TGF‐β, which has been described as highly labile, with a half‐life of the order of minutes [37]. The morphological analyses were achieved after a 35‐min treatment with GDF15 (4 nM).

The animal study protocol was in accordance with the guidelines of the European Communities Council Directive 2010/63/UE and approved by the Institutional Animal Care Committee (University of Florence, Italy), subjected to the authorisation of the Italian Ministry of Health (0DD9B.N.B9D/2024) for mice and (1114/2020‐PR) for rats.

### 2.2 Western Blotting

Murine gastric samples (CTRL) were dispersed in a buffer containing 50 mM Tris, pH 7.5, 120 mM NaCl, 1 mM EDTA, 6 mM EGTA, 15 mM Na_4_P_2_O_7_, 20 mM NaF, 1% Nonidet and protease inhibitor cocktail and disrupted in a Dounce homogeniser (100 strokes). Lysates were obtained following centrifugation (10,000× g for 15 min at 4°C) and resuspended in Laemmli sample buffer (Bio‐Rad Laboratories, Hercules, CA, USA) before being loaded on 10% SDS‐PAGE gels (Bio‐Rad Laboratories) and blotted onto PVDF membranes. Primary antibodies, rabbit polyclonal anti‐FRAL (1:1000; 43,96 kDa, Biorbyt, Durham Cat # orb32651, Lot#N7323, RRID AB_10939859) or mouse monoclonal anti‐RET (1:500; 50/55 kDa, Santa Cruz Biotechnology, Cat # sc‐101,423, Lot# I1622, RRID AB_2269604) were added to the PVDF membranes overnight at 4°C, while the horseradish peroxidase‐conjugated secondary antibodies (1:5000; Santa Cruz, CA, USA) were added for 1 h at room temperature. Enhanced chemiluminescence reagent (GE Healthcare Europe (Milan, Italy)) was employed to detect immunoreactive bands by Amersham Imager 600 (GE Healthcare, Chicago, IL, USA).

### 2.3 Electrophysiological Recordings

The rodent muscle strips from the gastric fundus were bathed with Krebs–Henseleit (KH) solution having the following composition (mM): 118 NaCl, 4.7 KCl, 1.2 MgSO_4_, 1.2 KH_2_PO_4_, 25 NaHCO_3_, 2.5 CaCl_2_, and 10 glucose (pH 7.4). Each strip was then pinned in the recording chamber (Petri dish p35 coated with a layer of Sylgard) with the external longitudinal muscle layer facing upwards. Intracellular recordings were achieved by a conventional high‐resistance glass microelectrode introduced into a SMC as described in previous studies [24, 38]. The microelectrodes were generated from borosilicate glass tubes (GC150‐7.5, Harvard apparatus LTD) using a two‐stage vertical puller (Narishige, Tokyo, Japan). They were normally filled with the intracellular solution (mM): 130 KCl, 10 NaHPO_4_, 0.2 CaCl_2_, 1 ethylene‐bis (oxyethylenitrilo) tetraacetic acid (EGTA), 5 MgATP and 10 4‐(2‐hydroxyethyl)‐1‐piperazineethanesulfonic acid (HEPES)/KOH. Once filled, the resistance of the pipette was 60–70 MΩ. The pH was set to 7.4 with NaOH and to 7.2 with tetraethyl ammonium hydroxide for the bath and pipette solution, respectively. All of the chemicals were obtained from Sigma‐Aldrich. The patch pipette was inserted in a CV203BU head‐stage (Axon Instruments, Foster City, CA) attached to a three‐way coarse manipulator and micro‐manipulator (Narishige, Tokyo, Japan) and an Axopatch 200 B amplifier (Axon Instruments, Foster City, CA) as previously described [39]. The generation of current‐ and voltage‐clamp protocols as well as data acquisition were accomplished by the A/D‐D/A interfaces (Digidata 1200; Axon Instruments) and Pclamp 6 software (Axon Instruments Foster City, CA). To record the resting membrane potential (RMP) of the SMCs before and after chemical treatments, we used the current‐clamp mode of the amplifier with a pulse waveform of I = 0 pA. To evaluate the membrane passive properties of SMCs, that is, resting membrane resistance (Rm) and cell capacitance (Cm), we operated in voltage clamp condition by applying two voltage steps of 75 ms, from −80 to −60 mV starting from a holding potential (HP) of −70 mV.

### 2.4 Morphological Analyses

#### 2.4.1 Haematoxylin and Eosin

Gastric fundus muscle strips from mice (*n* = 5) and from rats (*n* = 3) were fixed with 10% buffered formalin (Sigma‐ Aldrich Corp), dehydrated with a graded alcohol series, cleared in xylene, and embedded in paraffin [24]. At least ten 5 μm‐thick sections were cut from each sample (mouse: *n* = 50, rat: *n* = 30—for each experimental condition). At least 10 sections for each experimental condition were routinely stained with Haematoxylin and Eosin (H&E) (both Bio‐Optica, Milan, Italy), following standard histological protocols. Tissue morphology was observed under a light microscope (Leica DM4000 B) equipped with a DFC310 FX 1.4‐megapixel digital colour camera and software application suite LAS V3.8 (Leica Microsystems, Mannheim, Germany).

#### 2.4.2 Confocal Laser Scanning Microscopy

Confocal Laser Scanning Microscopy (CLSM) analyses were performed on paraffin embedded muscle strip 5 μm‐thick sections (at least 10 sections for each experimental condition) following previously reported procedures [24]. For indirect single or double immunofluorescence, the following primary antibodies were employed (incubated overnight at 4°C): rabbit polyclonal anti‐GFRAL (1:50; Biorbyt); mouse monoclonal anti‐RET (1:100; Santa Cruz Biotechnology); goat polyclonal anti‐Choline Acetyltransferase (ChAT, 1:300, Merck Sigma‐Aldrich, Cat#AB144P, Lot# 4010668, RRID:AB_2079751) mouse monoclonal anti‐α‐smooth muscle actin (sma) (1:100; Abcam, Cambridge, UK, Cat #ab7817, Lot# GR3246513, RRID AB_262054); and rabbit anti‐phospho‐myosin light chain (p‐MLC)‐2 (Ser‐19) (1:100; Cell Signalling Technology, Danvers, MA, USA, Cat #3671, Lot #7, RRID AB_330248). The immunoreactions were revealed by using an anti‐mouse Alexa Fluor 488‐conjugated IgG (1:200; 1 h at room temperature, Molecular Probes‐Thermo Fisher Scientific, Eugene, OR, USA, Cat # A11001, Lot# 1752514, RRID AB_2534069) or anti‐rabbit Alexa Fluor 488‐conjugated IgG (1:200, 1 h at room temperature, Molecular Probes‐Thermo Fisher Scientific, Cat #A11034, Lot# 1094393 RRID AB_2576217) or anti‐goat Alexa 568‐conjugated IgG (1:200, 1 h at room temperature, Molecular Probes‐Thermo Fisher Scientific, Cat#A11079, Lot#1148366, RRID:AB_2534123). Negative controls were attained by replacing the primary antibodies with non‐immune serum, and the cross‐reactivity of the secondary antibodies was evaluated by omitting the primary ones. Nuclei were counterstained with Propidium iodide (PI, Molecular Probes, Cat # P1304MP; 1:100, at room temperature for 2 min) or 4′, 6‐diamidino‐2‐phenylindole (DAPI, Sigma‐Aldrich, Cat#MBD0020, 1:1000 at room temperature for 10 min). Samples were observed with a confocal microscope Leica TCS SP5 equipped with a HeNe/Ar laser source for fluorescence measurements and differential interference contrast (DIC) optics by means of an objective Leica Plan Apo 40xNA or with a Leica Stellaris 5 confocal microscope equipped with a white light source for fluorescence measurements by using a Leica Plan Apo 63×/1.43NA oil immersion objective (Leica Microsystems, Mannheim, Germany). Optical section series (1024 × 1024 pixels each, pixel size 204.3 nm, 209 × 209 μm optical square field) were acquired every 0.4 or 0.6 μm and projected onto a single ‘extended focus’ image. Differential interference contrast (DIC) images were simultaneously acquired with fluorescence images. 3D viewer was performed by Leica Application Suite X (LAS X 3D‐ The User interface of the 3D Viewer).

Digitised images were subjected to densitometric analysis of α‐sma and p‐MLC2 fluorescent signal intensity (FI) by using ImageJ software (Version 1.49 v, RRID:SCR_003070; NIH, Bethesda, MD, USA). For each confocal stack (three for each experimental condition/each animal) five regions of interest (ROI; 25 × 25 μm) were analysed (ROI = 75 mouse; ROI = 45 rat).

### 2.5 Statistical Analysis

The statistical analysis of the results was carried out with Microsoft Excel (Microsoft, Washington, USA). To assess the normal distribution of data, we used the Shapiro–Wilk test. Two‐tailed Student's *t*‐test was used to compare the means of two data sets, and the one‐way ANOVA with Bonferroni post hoc correction for multiple comparisons. Values are reported as mean ± SD. Statistical significance was set at *p* < 0.05. Microsoft Excel was used to generate graphs.

## 3 Results

### 3.1 GDF15 Affects Resting Membrane Potential (RMP) and Contractile Apparatus of Murine Gastric Fundus SMCs

**FIGURE 1:**
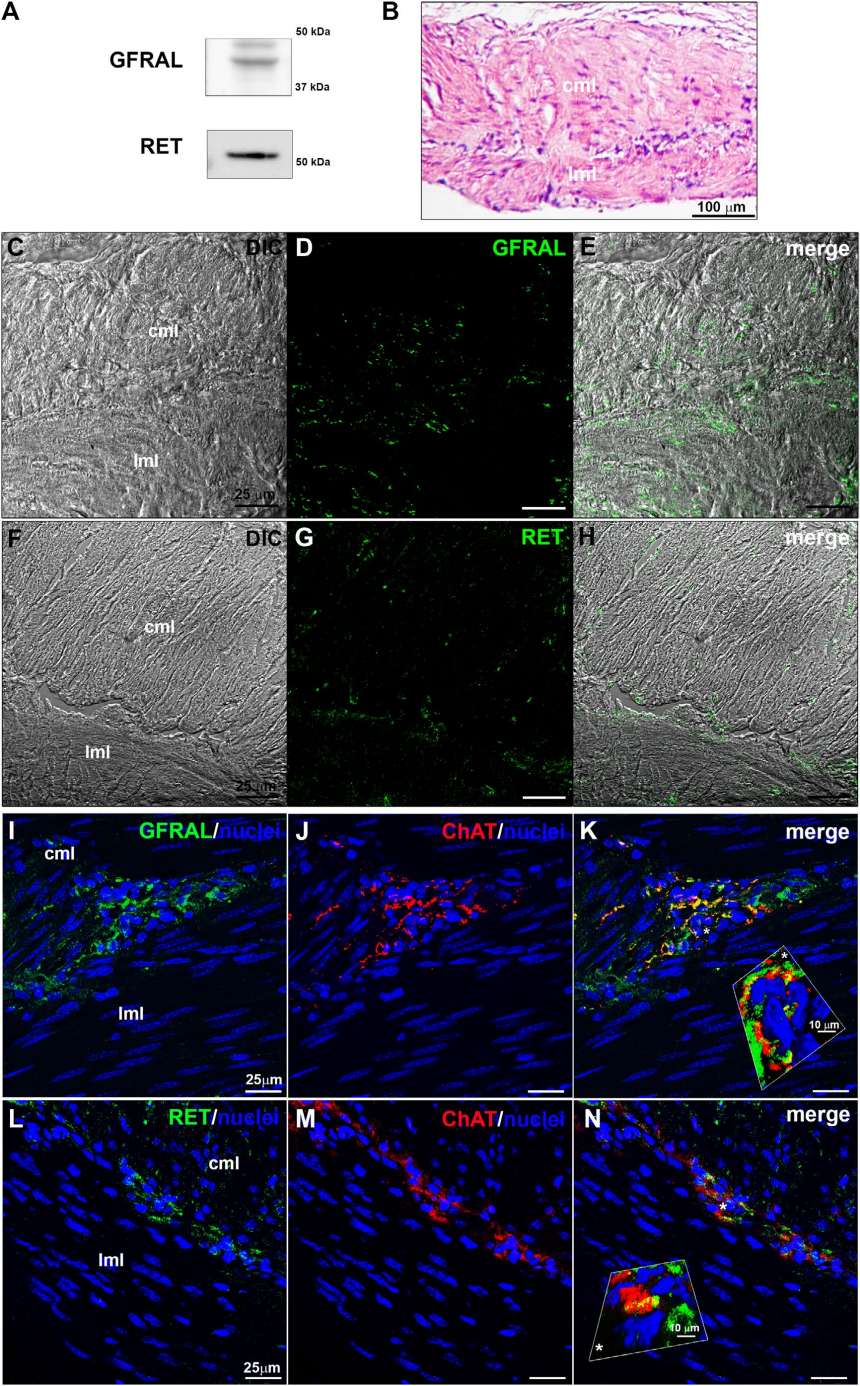
Expression and localization of GFRAL and RET in murine gastric fundus muscle strips. (A) Western blot analysis performed in murine gastric fundus smooth muscle tissue lysates using specific antibodies against GFRAL or RET. (B) Representative light microscopy image of a longitudinal section of formalin‐fixed and paraffin‐embedded muscle strip from control (CTRL) gastric fundus stained with haematoxylin and eosin (H&E; cml = circular muscle layer, lml = longitudinal muscle layer). (C–N) Representative confocal laser scanning microscopy (CLSM) images. (C, F) Differential interference contrast images (DIC, grey) and (D, E, G, H) relative confocal immunofluorescence images of muscle strip immunostained with antibodies against GFRAL (D, green) or RET (G, green) acquired simultaneously; in E and H superimposed DIC and images. (I–N) Double immunolabelling for GFRAL (I, green) or RET (L, green) and ChAT (J, M, red). Nuclei are counterstained in blue with DAPI. K and N: Merge of green and red fluorescent signals that highlights the co‐expression of GFRAL and RET in the cholinergic neurons of the myenteric plexus. Insets (*) in K and N exhibit a 3D view (Leica Application Suite X).

In order to define the effects of GDF15 on gastric fundus muscle layer, we first determined whether the receptor for GDF15, namely GFRAL and its co‐receptor RET, were expressed in our samples. Western blotting analysis performed in CTRL tissue lysates demonstrated that both GFRAL and RET were expressed (Figure 1A).

The CLSM immunofluorescence analysis of these proteins confirmed their expression in both external longitudinal and internal circular muscle layers (Figure 1C–H).

Moreover, in order to evaluate the expression of the receptor of GDF15 and of itsco‐receptor in cholinergic neurons, we performed a double immunolabelling for ChAT and GFRAL or RET (Figure 1I–N). The results unravelled the expression of both GFRAL (Figure 1I–K) and RET (Figure 1L–N) in the neurons of the myenteric plexus typically lying in between the outer longitudinal and inner circular smooth muscle layers of the gastric wall.

These results clearly demonstrate for the first time the expression of GFRAL and of its co‐receptor RET in murine gastric fundus muscle wall including the cholinergic neurons of the myenteric plexus.

**FIGURE 2:**
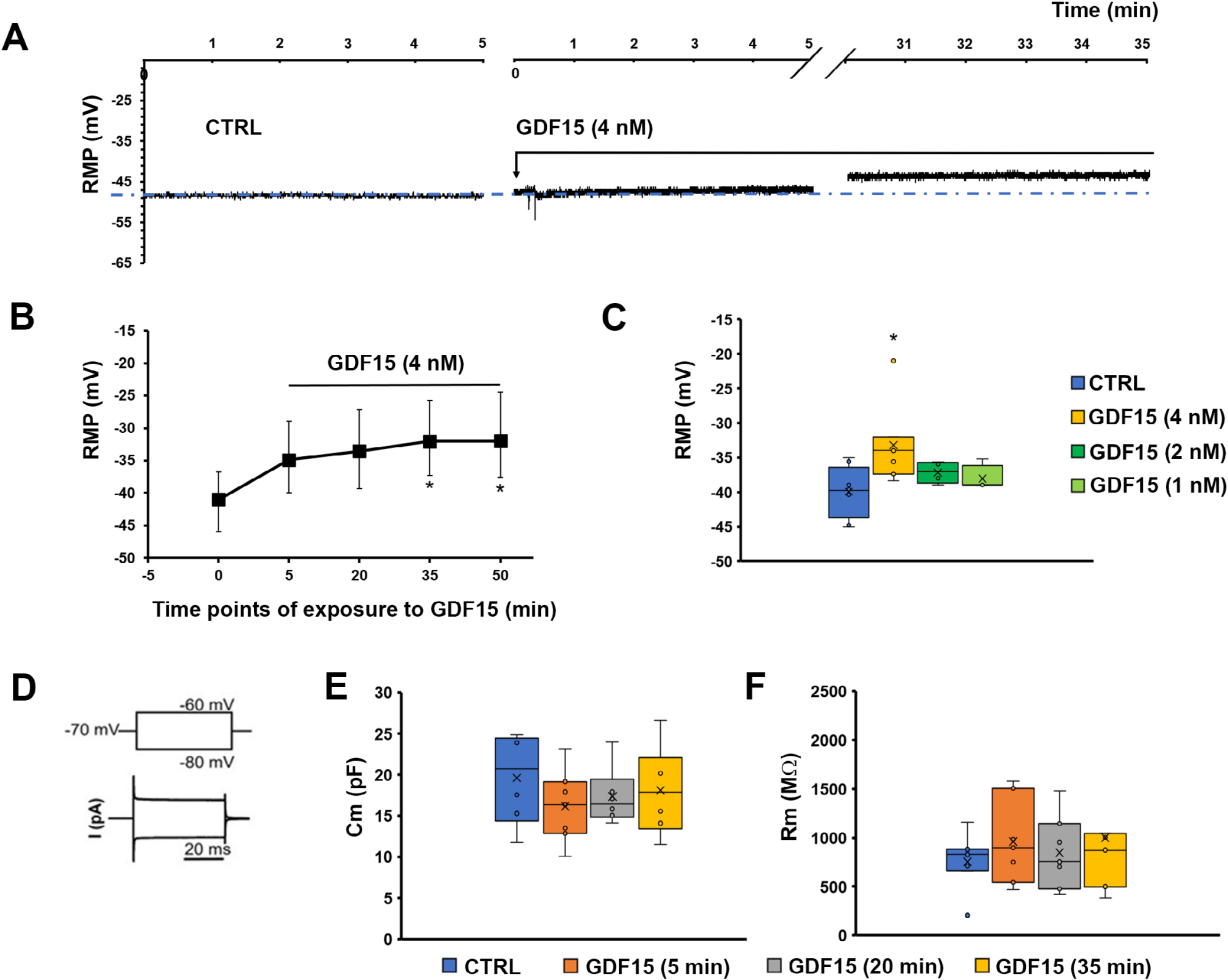
Effect of GDF15 on the membrane of smooth muscle cells (SMCs) in murine gastric fundus. (A) Representative time course of the resting membrane potential (RMP, in mV) of a murine SMC in control conditions (CTRL, left panel), after GDF15 (4 nM) addition (indicated by the arrow, middle panel), up to 35 min of exposure (right panel). Note that the *x*‐axis is interrupted for space reasons. The blue dash dot line indicates the resting membrane potential of the untreated cell. (B) Time‐dependent effect of GDF15 on RMP evaluated from all the experiments done in murine samples. (C) Effect of GDF15 used at progressively halved concentrations (4 nM, 2 nM, 1 nM) evaluated on RMP values after 35 min of exposure. RMP values (in mV) are −39.9 ± 3.6 for CTRL (*n* = 8); −33.2* ±5.8 for GDF15 (4 nM) (*n* = 7); −37.1 ± 1.6 for GDF15 (2 nM) (*n* = 4); −38.5 ± 1.9 for GDF15 (1 nM) (*n* = 4). (*p* = 0.03, one‐way ANOVA with Bonferroni post hoc correction). Only values obtained with GDF15 (4 nM) are significantly different compared with CTRL. (D): V‐clamp step pulse protocol of stimulation and representative passive current response. Effect of GDF15 (4 nM) on cell capacitance, Cm (E) and membrane resistance, Rm (F) in SMCs of gastric fundus estimated at different times of exposure. Passive parameter values and *n* related to the different times of exposure are listed in Table 1. Values are mean ± SD. *p < 0.05 versus CTRL. One‐way ANOVA with Bonferroni post hoc correction (*p*‐values listed in Table 1).

To test the potential action of GDF15 on SMC excitability, we measured the RMP in mouse samples (see Methods section on current clamp mode and relative devices). GDF15 (4 nM) caused RMP depolarization detectable after 5 min (Figure 2A). RMP was then measured at 15‐min intervals, showing that this effect kept on increasing with time, plateauing at around 30 min (Figure 2A,B). In fact, a statistically significant depolarization compared to values from CTRL samples was reached after 35 min of exposure and did not further change at longer times (50 min; Figure 2A). Consequently, 35 min were assumed as the time of exposure for GDF15's effect to plateau and be detected with a statistically significant effect on cell membranes. Accordingly, this time of exposure was also used in subsequent experiments. All recorded parameters and related statistical significance are listed in Table 1. In addition, when GDF15 concentrations were progressively halved to 2 and 1 nM to test the eventual efficacy of lower doses, no statistically significant alterations were observed compared with CTRL (Figure 2C; RMP values, *n*, and *p*‐values are listed in the legend of Figure 2). For this reason, we did not test doses lower than an order of magnitude, such as 0.1 nM or minor.

**TABLE 1:** Effect of GDF15 addition on membrane passive properties of rodent gastric fundus smooth muscle cells (SMCs).

	CTRL	GDF15 (4 nM)				*p*
	Baseline	5 min	20 min	35 min	50 min	
MOUSE						
RMP (mV)	−41.0 ± 4.2 (*n* = 7)	−34.8 ± 5.9 (*n* = 6)	−33.6 ± 6.4 (*n* = 6)	−32.0 ± 6.3* (*n* = 6)	−32.0 ± 7.5* (*n* = 5)	0.04
Cm (pF)	19.6 ± 5.5 (*n* = 6)	16.1 ± 4.4 (*n* = 7)	17.3 ± 3.5 (*n* = 6)	18.1 ± 5.4 (*n* = 6)	/	0.62
Rm (MΩ)	754.3 ± 289.9 (*n* = 7)	961.2 ± 435.3 (*n* = 7)	847.3 ± 375.8 (*n* = 7)	1000.1 ± 776.5 (*n* = 7)	/	0.79
RAT						
RMP (mV)	−49.3 ± 4.9 (*n* = 7)	−46.8 ± 5.1 (*n* = 5)	−44.8 ± 5.7 (*n* = 5)	−41.8 ± 5.3* (*n* = 5)	−40.4 ± 5.5* (*n* = 5)	0.04
Cm (pF)	27.9 ± 10.7 (*n* = 9)	30.2 ± 11.3 (*n* = 9)	33.1 ± 9.8 (*n* = 9)	33.1 ± 7.4 (*n* = 9)	/	0.64
Rm (MΩ)	1437.5 ± 541.6 (*n* = 6)	1573.3 ± 221.5 (*n* = 7)	2039.1 ± 536.0 (*n* = 7)	1949.9 ± 869.8 (*n* = 7)	/	0.22

*Note:* Values are means ± SD. Comparisons made between GDF exposure and control conditions, by one‐way ANOVA with Bonferroni post hoc correction.

Abbreviations: Cm, membrane capacitance; CTRL, control (untreated); GDF15, growth differentiation factor 15; *n*, number of SMCs; Rm, membrane resistance; RMP, resting membrane potential.

* *p* < 0.05 versus CTRL.

The effect of GDF15 was tested also on the membrane passive properties of gastric SMCs (i.e., Cm and Rm) by applying a suitable voltage step pulse protocol (Figure 2D). No significant alterations of Cm compared to CTRL were observed after 35 min from GDF15 (4 nM) addition in murine samples (Figure 2E). Similar results applied to Rm (Figure 2F). All the recorded parameters, the number of investigated cells (*n*) and the *p*‐value calculated from ANOVA are listed in Table 1.

These results indicate that GDF15 influences SMC excitability within the gastric fundus of mice, causing a striking RMP depolarisation without considerably altering other biophysicalmembrane passive properties.

**FIGURE 3:**
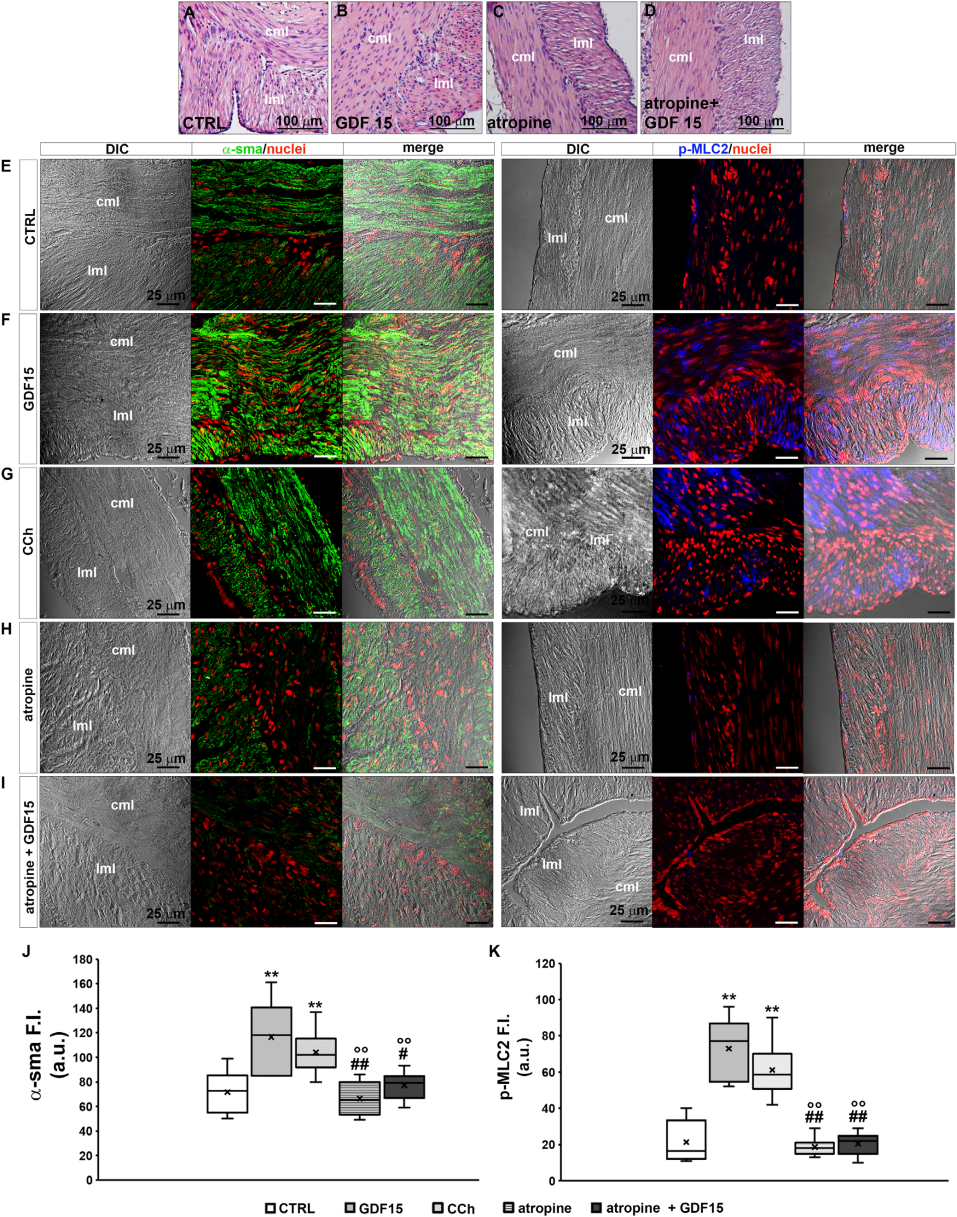
Morphological analyses of murine samples. (A–D) Representative light microscopy images of cross‐sectional cut formalin‐fixed and paraffin‐embedded muscle strips from murine control conditions (CTRL), GDF15‐treated (4 nM, 35 min), atropine‐treated (1 μM, 30 min) and atropine+GDF15 co‐treated (35 min) murine gastric fundus stained with haematoxylin & Eosin (H&E; cml, circular muscle layer; lml, longitudinal muscle layer). Atropine is the cholinergic antagonist used as a negative control of smooth muscle cell, SMC, contraction. (E–I) Confocal laser scanning microscopy (CLSM). Representative differential interference contrast images (DIC, grey) and related CLSM immunofluorescence images (acquired simultaneously) of formalin‐fixed and paraffin‐embedded muscle strip cross‐sections in the indicated experimental conditions, immunostained with antibodies against α‐sma (green) or against p‐MLC2 (blue, pseudocolor) and counterstained with propidium iodide for labelled nuclei (red); merge shows superimposed DIC and fluorescence images. CCh: Carbachol (cholinergic agonist used as positive control of muscle cell contraction, 1 μM, 35 min). (J, K). Quantitative analyses of α‐sma and p‐MLC2 fluorescence intensity (F. I.) signals in arbitrary units (a.u.). **p < 0.01 versus CTRL; °°*p* < 0.01 versus GDF15; #*p* < 0.05, ##*p* < 0.01 versus CCh. One‐way ANOVA with Bonferroni post hoc correction.

Since membrane depolarization can favour electromechanical coupling leading to SMC contraction, a morphological analysis was carried out to test whether GDF15 could also affect the contractile apparatus and related proteins. Although light microscopy analysis of H&E stained sections from CTRL and GDF15‐treated murine muscle strips did not reveal substantial structural differences between the samples (Figure 3A,B), CLSM immunofluorescence analysis of contractile proteins revealed suggestive features of GDF15‐related effects in the two different experimental conditions. Indeed, the analysis of the expression and organisation of α‐sma, the predominant actin isoform composing the thin myofilaments of the contractile machinery within SMCs showed that GDF15 enhanced the expression of this protein as compared to CTRL (Figure 3E,F,J). The more intense immunostaining of α‐sma observed in GDF‐treated samples may be consistent with the myofiber cytoplasmic organisation of such protein in “thick” aggregates (hence higher fluorescent signal), suggestive of the typical arrangement of the thin myofilaments in SMCs in a contracted state. In parallel, GDF15 treatment induced an augment of the expression of p‐MLC2, which is correlated with myosin ATPase activity and SMC contraction (Figure 3E,F,K).

The expression of both proteins appeared comparable to that observed in the samples treated with CCh, the cholinergic agonist used as a positive control of SMC contractile response (Figure 3E–GJ,K).

### 3.2 GDF15‐Induced Effects on Murine Gastric Fundus SMCs Are Hampered by the Treatment With the Cholinergic Muscarinic Receptor Antagonist Atropine

**FIGURE 4:**
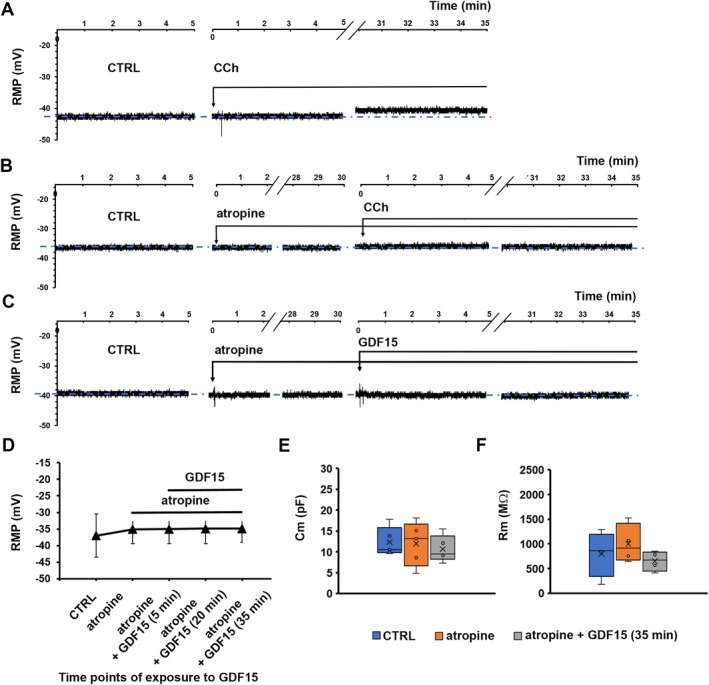
Abolition of the depolarizing effect of carbachol (CCh) and of GDF15 by atropine pre‐treatment on murine gastric smooth muscle cells (SMCs). (A) Representative time course of the resting membrane potential (RMP, in mV) of a murine SMC in control conditions (CTRL, left), after CCh application (1 μM) (indicated by the arrow, middle), and in the presence of CCh up to 35 min of exposure (right). (B) Representative time course of the RMP of a murine SMC in CTRL condition (left), after atropine application (1 μM) (indicated by the arrow, middle left) for 30 min and after CCh added in the presence of atropine (up to 35 min of concomitant exposure, right). (C) Representative time course of the resting membrane potential (RMP, in mV) of a SMC in control condition (CTRL, left panel), after atropine application (1 μM, 30 min‐pretreatment) (indicated by the arrow, middle panel), and with GDF15 (4 nM) applied in the concomitant presence of atropine, recorded for 35 min of concomitant exposure (right panel). Note that the *x*‐axis is interrupted for space reasons. The blue dash dot line indicates the resting membrane potential of the untreated cell. (D) Lack of effects of GDF15 on RMP, when the treatment was made in the concomitant presence of atropine, evaluated from all the experiments done in mice. (E, F) Effect of atropine alone (1 μM, 30 min) and in the presence of GDF15 (4 nM, 35 min) on cell capacitance, Cm (E) and membrane resistance, Rm (F) in murine SMC. The recorded parameters and the number of investigated cells (*n*) are listed in Table 2. Values are mean ± SD. *p* > 0.05. One‐way ANOVA with Bonferroni post hoc correction (*p*‐values listed in Table 2).

Having consistently observed that GDF15 induced a membrane depolarization in our samples, and since this depolarization was associated with cues of activation of the contractile machinery, we hypothesised a possible involvement of the cholinergic stimulus in its mechanism of action, given the resemblance of observed results with those usually mediated by M(3) receptors [40]. Consistent with this, the treatment with the muscarinic receptor agonist CCh (1 μM; 35 min) not only induced morphological alteration in terms of expression of contractile proteins (Figure 3G,J,K) but also functional alterations typical of cells in an active status of contraction (Figure 4A). In particular, the RMP was recorded in 15‐min intervals after the addition of CCh to the bath medium up to 50 min, in line with GDF15 experiments. The results highlighted that the membrane became indeed more positive (Figure 4A), reaching an overall depolarization after 35 min that remained stable for a longer time interval (50 min, not shown). Confirming this, when CCh was added in the presence of the cholinergic muscarinic antagonist atropine (1 μM) after a 30 min pre‐treatment, the RMP remained unaltered, endorsing the involvement of the muscarinic receptor in causing the membrane excitatory effect (Figure 4B).

Then, we performed functional and morphological analyses of the sample treated with atropine (1 μM) alone and in the concomitant presence of GDF15 (4 nM). A 30 min exposure to atropine alone did not cause significant changes either in the morphological features and contractile apparatus protein expression (Figure 3H,J,K) or in RMP values (Figure 4C,D) as compared to CTRL. After atropine pre‐treatment, GDF15 was added in the same bath medium containing the muscarinic receptor antagonist. Again, records were achieved after 5 min from GDF15 addition and then at 15 min intervals, up to 35 min of simultaneous presence. We found that atropine negated GDF15‐related RMP depolarization of the SMC membrane (Figure 4C) and this result was consistently confirmed across all time‐points under evaluation (Figure 4D). Concurrently, morphological analyses endorsed the interpretation that GDF15‐related pro‐contractile effects on SMC involve muscarinic receptors (Figure 3I–K): in fact, in the concomitant presence of atropine, GDF15 was not even capable of significantly increasing α‐sma (Figure 3E,F) or p‐MLC2 (Figure 3E,F,K) compared to CTRL.

Finally, we confirmed that GDF15 was not able to alter significantly Cm and Rm of the SMC membrane, regardless of the presence of atropine (Figure 4C,D). All of the resulting parameters and *p*‐values calculated from ANOVA are listed in Table 2 and are in line with previously described morphological features, which showed no structural cellular alterations caused by GDF15, atropine, or the co‐treatment with atropine and GDF15 (Figure 3B,C).

**TABLE 2:** Effect of GDF15 addition in the presence of atropine on membrane passive properties of rodent gastric fundus smooth muscle cells (SMCs).

	CTRL	Atropine	Atropine + GDF15			*p*
	Baseline	30 min	5 min	20 min	35 min	
MOUSE						
RMP (mV)	−36.97 ± 6.5 (*n* = 6)	−35.07 ± 4.3 (*n* = 6)	−34.9 ± 4.4 (*n* = 6)	−34.9 ± 4.4 (*n* = 6)	−34.9 ± 4.1 (*n* = 6)	0.93
Cm (pF)	12.3 ± 3.5 (*n* = 5)	11.9 ± 5.2 (*n* = 5)	/	/	10.7 ± 3.2 (*n* = 5)	0.79
Rm (MΩ)	796.5 ± 462.6 (*n* = 4)	998.1 ± 396.3 (*n* = 5)	/	/	651.4 ± 203.9 (*n* = 5)	0.44
RAT						
RMP (mV)	−39.1 ± 2.9 (*n* = 4)	−37.8 ± 2.7 (*n* = 4)	−37.8 ± 2.7 (*n* = 4)	−37.8 ± 2.8 (*n* = 4)	−37.8 ± 2.5 (*n* = 4)	0.95
Cm (pF)	20.7 ± 10.7 (*n* = 3)	17.6 ± 11.9 (*n* = 3)	/	/	18.6 ± 14.1 (*n* = 3)	0.95
Rm (MΩ)	1258.6 ± 537.8 (*n* = 4)	1842.6 ± 504.1 (*n* = 4)	/	/	1951.4 ± 1023.8 (*n* = 4)	0.39

*Note:* Values are means ± SD. Comparisons made versus CTRL by one‐way ANOVA with Bonferroni post hoc correction.

Abbreviations: Cm, membrane capacitance; CTRL, control (untreated); GDF15, growth differentiation factor 15; *n*, number of SMCs; Rm, membrane resistance; RMP, resting membrane potential.

In summary, GDF15 added in the presence of atropine lost its ability to depolarize the SMC membrane in mice, without causing further alterations of membrane passive properties. Similarly, GDF15 lost its ability to increase the markers of contractility, suggesting a more relaxed condition under the concomitant atropine treatment.

### 3.3 Cross‐Species (Rat) Confirmatory Analyses

**FIGURE 5:**
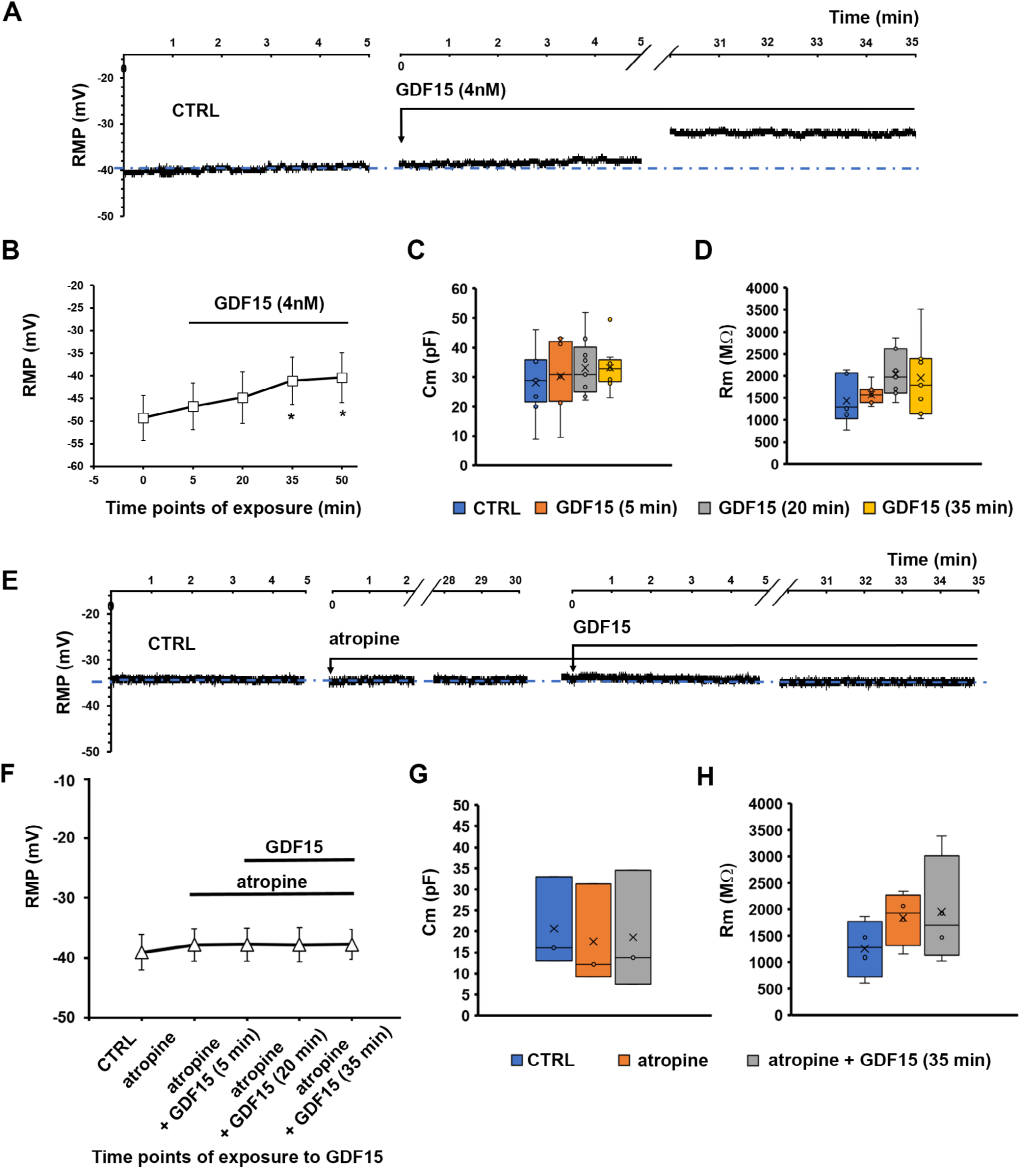
Effect of GDF15 on the membrane of rat gastric fundus smooth muscle cells (SMCs) and its abolition by atropine pre‐treatment. (A) Representative time course of the resting membrane potential (RMP, in mV) of a rat SMC in control conditions (CTRL, left panel), after GDF15 (4 nM) application (indicated by the arrow, middle panel), up to 35 min of exposure (right panel). Note that the *x*‐axis is interrupted for space reasons. The blue dash dot line indicates the resting membrane potential of the untreated cell. (B) Time dependent effect of GDF15 on RMP evaluated from all the experiments done in rat preparations. Values are mean ± SD (Table 1). *p < 0.05 versus CTRL. One‐way ANOVA with Bonferroni correction (*p*‐values listed in Table 1). (C, D) Effect of GDF15 on cell capacitance, Cm (C) and membrane resistance, Rm (D) estimated at different times of exposure. Passive parameter values and *n* related to the different times of exposure are listed in Table 1. *p* > 0.05 versus CTRL. One‐way ANOVA with Bonferroni correction (related *p*‐values in Table 1). (E) Representative time course of the resting membrane potential (RMP, in mV) of a rat SMC in control conditions (CTRL, left panel), after atropine application (1 μM) (indicated by the arrow, middle panel), and with GDF15 (4 nM) applied in the concomitant presence of atropine, recorded for 35 min of exposure (right panel). Note that the *x*‐axis is interrupted for space reasons. (A) and (E) show RMP from two different SMCs. The blue dash dot line indicates the resting membrane potential of the untreated cell. (F) Lack of statistically significant effects of GDF15 on RMP, when the treatment was made in the concomitant presence of atropine, evaluated from all the experiments done in rats. (G, H) Effect of atropine (1 μM, 30 min) alone and in the presence of GDF15 (4 nM) on cell capacitance, Cm (G) and membrane resistance, Rm (H) in SMCs estimated after 35 min of exposure to GDF15: Parameter values (as mean ± SD) and *n* are listed in Table 2. *p* > 0.05. One‐way ANOVA with Bonferroni correction (*p*‐values listed in Table 2).

Experiments conducted on SMCs of rat gastric fundus confirmed the observations obtained in mice. Indeed, GDF15 (4 nM) induced a statistically significant RMP depolarization of SMC membrane compared to CTRL after 35 min of treatment (Figure 5A,B), whereas the membrane passive properties were not significantly altered (Figure 5C,D and Table 1).

**FIGURE 6:**
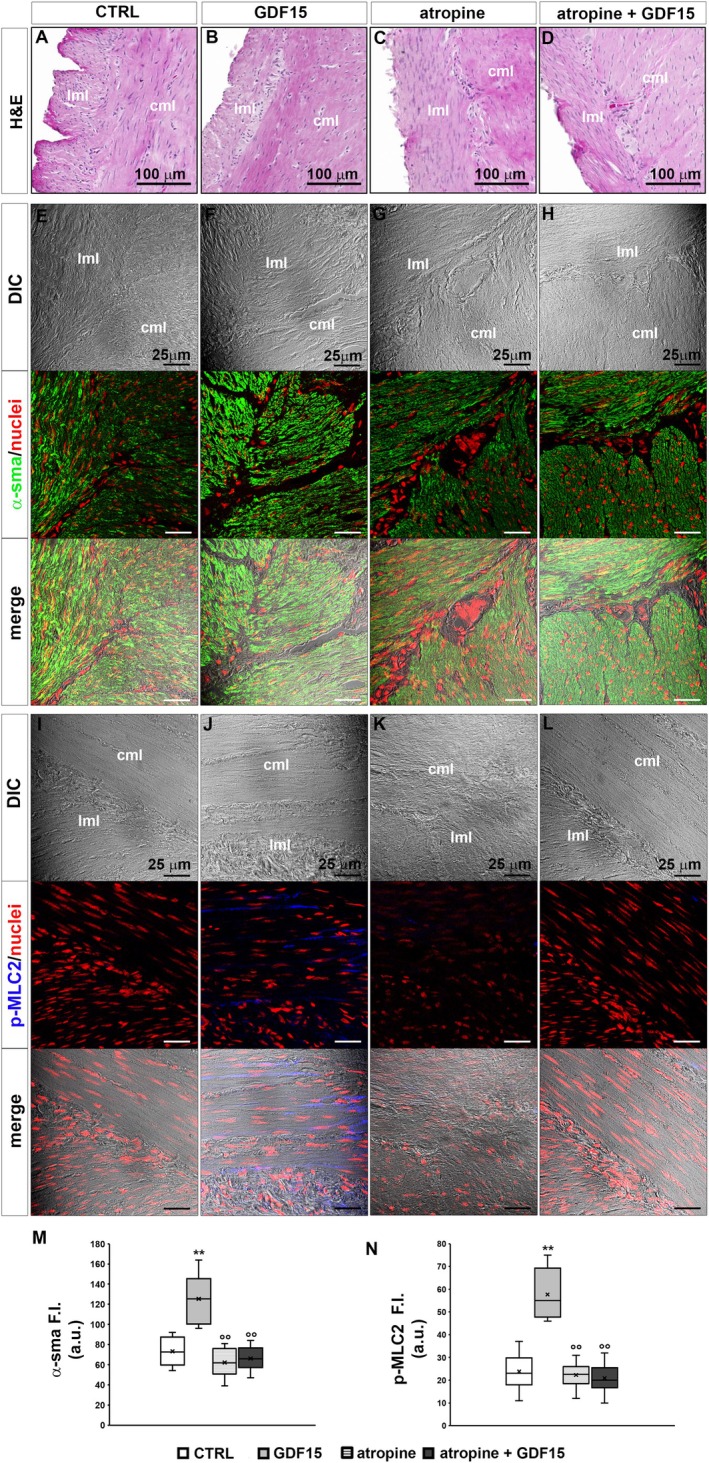
Morphological analyses of rat samples. (A–D) Representative light microscopy images of cross‐sectional cut formalin‐fixed and paraffin‐embedded muscle strips from control conditions (CTRL), GDF15 (4 nM)–treated, atropine (1 μM)‐treated and atropine+GDF15 co‐treated rat gastric fundus, stained with haematoxylin and eosin (H&E, cml = circular muscle layer, lml = longitudinal muscle layer). (E‐L) Confocal laser scanning microscopy (CLSM). Representative Differential interference contrast images (DIC, grey) and relative confocal immunofluorescence images (acquired simultaneously) of muscle strips, immunostained with antibodies against (E‐H, longitudinal sections) α‐sma (green) or (I‐L, transverse sections) p‐MLC2 (blue, pseudocolor) and counterstained with propidium iodide for label nuclei (red); merge shows superimposed DIC and fluorescence images. (M, N) quantitative analysis of α‐sma and p‐MLC2 fluorescence intensity (F.I.) signals in arbitrary units (a.u.). **p < 0.01 versus CTRL; °°*p* < 0.01 versus GDF15. One‐way ANOVA with Bonferroni post hoc correction.

Consistent with the electrophysiological data of passive properties, light microscopy images of rat gastric fundus muscle strips stained with H&E did not show appreciable structural modifications between CTRL and GDF15‐treated samples (Figure 6A,B). However, as observed in mouse samples, GDF15's depolarizing effect on RMP was accompanied by a clear increase of α‐sma (Figure 6E,F,M) and p‐MLC2 (Figure 6E,I,J,N) suggesting again a pro‐contractile effect of GDF15 also on rat gastric fundus SMCs. Likewise, the involvement of cholinergic signals in GDF15 effects was confirmed in rat gastric fundus samples. Indeed, while the atropine treatment alone did not induce appreciable functional (Figure 5E,F) or morphological effects compared to CTRL (Figure 6C,G,K,M,N), the atropine pre‐treatment for 30 min hindered the GDF15‐induced depolarization of SMC membrane (Figure 5E,F) as well as the increase of the analysed contractile markers (Figure 6H,L–N). Similar to what was observed in the mouse, also in the rat, GDF15 was not able to alter significantly Cm and Rm of the SMCs regardless of the presence of atropine (Figure 5G,H). All of the resulting values are listed in Table 2 and are in line with the morphological features.

Overall, the results of our morpho‐functional analyses across two different rodent models indicate a pro‐contractile effect of GDF15 in the gastric fundus smooth muscle layer and suggest the involvement of cholinergic receptors as effectors of its action.

## 4 Discussion

GDF15 is a pleiotropic peptide involved in various physiological and pathological conditions [7, 41]. Notably, GDF15 may have a role in appetite regulation: it has been considered able to suppress food intake and determine loss of body weight, inducing nausea, vomiting and anorexia [1, 42, 43]. These anorexigenic and weight‐suppressive actions make GDF15 a very attractive molecule from a translational point of view, encouraging potential applications in the treatment of obesity, metabolic disorders, but also feeding and eating disorders [13, 44].

In this study, which encompasses the scope of anorexigenic actions through its interplay with peripheral gastric signals, we tried to elucidate the potential effects of GDF15 on the gastric smooth muscle using a multidisciplinary approach on ex vivo preparations from rodent models. Our aim was to test the possible impact of GDF15 in modulating peripheral anorectic signals originating from the stomach that may occur in conditions of stress, where its plasma levels are usually increased. As previously mentioned, GDF15 has been shown to increase as a function of age, with serum levels up to 2,152 pg/mL at age ≥ 80 years [11]. Moreover, GDF15 has been shown to be increased up to 19,311 pg/mL in pregnant women [11]. Accordingly, current results obtained with a high GDF15 concentration, corresponding to 4 nM, may be representative of conditions of clinical interest, showing potential implications for an interplay between circumstances where GDF15 has been shown as increased, and peripheral effects on the gastric fundus muscle layer (potentially contributing, at least in part, to systemic effects such as nausea, visceral malaise, and emesis). In fact, although the stomach can be recognised as the site of origin of pathological conditions such as gastritis, gastroparesis and vomiting, the gastric fundus is also regarded as a physiological site of origin for peripheral signals capable of modulating food intake [45, 46]. Indeed, vagal afferent fibres project centrally from the gastric wall muscle layer and myenteric plexus to the nucleus of the solitary tract (NTS) and from here to higher brain centers, ultimately leading to sensations such as satiety and nausea, as well as to behavioural responses such as an inhibition of food seeking behaviours [47, 48].

The smooth muscle layer of the stomach wall is responsible for generating the peristaltic contractions that mix and propel the gastric contents towards the pylorus and for the gastric basal tone. This is due to the continuous and passive partial contraction of the muscle layer which is essential for maintaining the organ shape. The basal tone, mainly due to the proximal stomach, is primarily regulated by the autonomic efferent nervous system, the enteric nervous system (myenteric plexus) and various humoral factors. With regard to the autonomic parasympathetic nervous control mediated by acetylcholine, the M(3) receptor is recognised as the major muscarinic receptor involved in smooth muscle contraction of the stomach fundus [40]. As well, a number of non‐cholinergic neurotransmitters and a variety of hormones like gastrin, motilin and adipokines take part in this complex mechanism [49, 50, 51].

Our current results show that in concentrations similar to those associated with pathological or para‐physiological conditions, GDF15 may exert an influence on gastric tone. The interest in the possible role of GDF15 in modulating gastric smooth muscle excitability and tone has recently increased, potentially linking elevated levels of GDF15 to appetite dysregulation, nausea and emetic responses [1]. GDF15 is in fact considered a hormone acting in the hindbrain that activates neural circuitry involved in establishing aversive responses in animal models. There is compelling evidence in rodents and non‐human primates that manipulation of the GDF15‐GFRAL pathway induces an anorectic effect [3, 4, 44]. At present, evidence supporting the anorectic potential of GDF15 in humans is largely limited to observational studies [52]. Therefore, at the moment, rodent models provide a valuable tool to investigate the structural and functional changes within the stomach that are otherwise challenging to study in humans.

Interestingly, we revealed for the first time the presence of GFRAL/RET in the gastric muscle wall taken from healthy mice, validating the idea that this peptide may also influence gastric physiology acting at a peripheral level. Of note, we also unravelled that both the GFRAL receptor and its co‐receptor RET were co‐expressed in the cholinergic neurons of the myenteric plexus. This peculiar receptor location/arrangement, apart from the well‐known described localisation in the brainstem of the central nervous system, corroborates previous observations: GFRAL transcripts, even very slight, have been already detected in the peripheral tissues, such as small intestine, of both humans and mice [5, 53]. Our results thus expand upon previous studies and clearly revealed GFRAL expression in the stomach, suggesting broader applicability in a translational perspective.

Overall, these findings ultimately substantiate the hypothesis that GDF15 exerts a pleiotropic role across a variety of tissues, both in physiological and pathological conditions. It has already been suggested that an upregulation of GFRAL may occur under stress or injury, such as obesity or specific developmental stages in mice and humans [54, 55]. Hence, it is of great interest to achieve a better characterisation of the interplay between cellular stress, tissue injury and GFRAL/RET upregulation, beyond GDF15 secretion alone [20].

To our knowledge, this is the first study that demonstrates a pro‐contractile action of GDF15. While this finding suggests several scenarios involving muscle tissue, such as cardiovascular or skeletal muscle, at the same time, it can change the assumption that the anorectic effect of GDF15 is given only by NTS/area postrema (AP) at a central level. The membrane depolarization and the increased expression of contractility markers observed in this study are suggestive of making gastric smooth muscle more prone to achieve a contractile response, and this may be rather surprising, considering the previously described anorectic effect of the peptide. In fact, based on our previous results obtained with another anorexigenic hormone on the same gastric region—adiponectin [24, 56]—anorexigenic effects have been previously described as mainly related to SMC hyperpolarisation and markers indicative of a more relaxed cell, thus favouring gastric fundus distension, predisposing to delayed gastric emptying and triggering signals of satiety and fullness to the brainstem and hypothalamus. Nonetheless, the pro‐contractile effect of GDF15 is not necessarily in contrast with the promotion of anorexia and weight loss. In fact, evidence has been reported on the association between impaired relaxation or increased excitation in the gastric fundus with nausea and satiety, potentially through quicker gastric emptying [25], food aversion, and a general feeling of malaise [1, 57]. More specifically, Borner and colleagues recently described that GDF15 is indeed capable of causing an aversive state associated with visceral malaise, reducing food intake [1]. This mechanism elicits a conditioned aversive response that leads to chronic, rather than acute, anorexia—where delayed gastric emptying is not a necessary condition for reduced food intake. The proposed pro‐contractile effect observed with a high GDF15 concentration may likely lead to an increased gastric smooth muscle tone and, therefore, to major internal pressure and/or impaired relaxation. This may contribute to functional dyspepsia and other motility disorders that could further cause visceral malaise and anorexia [1], as well as general functional gastrointestinal symptoms [58]. Experiments are ongoing in our laboratories to test GDF15 efficacy in other gastrointestinal tracts of murine models since a tissue‐specific action, as reported for other modulatory peptides, cannot be ruled out. In particular, investigations of gastric antrum response could be interesting and clarifying for the overall function of the stomach, since the antrum plays a significant role in gastric emptying.

Our data thus support an anorectic effect which can be promoted by visceral malaise rather than gastric fullness. To be noted, although GDF15 secretion is inhibited by fasting, these functional complaints are also frequently reported in restrictive eating [59]. These may reflect higher serum levels of GDF15 due to oxidative stress during fasting [60], for which lower GDF15 secretion in gastric tissue may only partly compensate. Alternatively, GFRAL/RET upregulation may be associated with pathophysiological alterations associated with restrictive eating. Future research may then be interested in clarifying the relationship between restrictive eating and the GDF15 axis, aiming at better understanding potential (mal)adaptive homeostatic responses involving central and peripheral tissues.

Of note, the present manuscript proposes a possible mechanism of action of GDF15 in the gastric wall. Indeed, our results robustly support the involvement of the cholinergic activity in mediating the pro‐contractile effect of GDF15. Nevertheless, further experiments are needed to describe and define the molecular mechanism of GDF15's action in detail. Interesting outcomes could come from electrical field stimulation (EFS) used to neurally activate gastric contraction [61], once the mechanism will be deeply clarified. However, at this stage of the research this stimulation practice would not have given us certainty about the mechanism underpinning the contractile modulating action of GDF15. Indeed, EFS simultaneously stimulates different types of myenteric plexus neurons that release either excitatory neurotransmitters (ACh, substance P) inducing contraction, or inhibitory mediators (nitric oxide, vasoactive intestinal peptide) inducing relaxation [49], thus leading to a complex and less predictable response and making it difficult to discriminate the specific role of the tested factor.

The membrane depolarization observed with GDF15, in fact, is a characteristic that can be generally observed in the SMCs of the proximal stomach wall as a consequence of cholinergic stimuli, usually mediated by M(3) receptors [40], which enhance the tonic contraction referred to as gastric tone [58]. Accordingly, both GDF15‐induced depolarization and the increase of markers of SMC contractility counteracted by the concurrent atropine treatment suggest the involvement of cholinergic signals in GDF15 mechanism of action. Nonetheless, in agreement with the detection of the GFRAL/RET within both the myenteric plexus and the smooth muscle layers of the gastric fundus wall, we can hypothesise a double action of GDF15 in stimulating the release of Ach either at the cholinergic neuron of the myenteric ganglia level or at the cholinergic nerve ending. In addition, we cannot rule out the possibility that GDF15 improves the efficiency of Ach action acting on the muscarinic receptors located on the SMCs. In fact, the ability to modify both the ligand‐recognition properties of these receptors on intestinal longitudinal muscle cells and the corresponding changes in longitudinal muscle contractility has already been reported for instance for TGF‐β, the same family of cytokines to which GDF15 belongs [62]. Based on the observed localisation of GDF15 receptor/coreceptor we cannot rule out that GDF15 acts in a dual manner, improving the efficiency of Ach on the SMCs on one hand and stimulating the release of Ach from cholinergic nerve endings on the other hand. In this case GDF15 could have an action similar to the gastrointestinal hormone motilin and its agonists [60, 63], activating gastric neuronal motilin receptors [64] or exerting a direct action on the muscle [65] with motilin receptors frequently being localised both in the smooth muscle and neurons located in the (human) gastric wall. Finally, enhanced cholinergic contraction of gastric smooth muscle after long term exposure to the molecule could even be achieved by augmenting muscarinic M(3) receptor expression, as previously reported for 7,8‐dihydroxyflavone in rat gastric smooth muscle [66].

Based on our findings, we can speculate that elevated levels of GDF15 observed in association with stress conditions like pregnancy, aging and exercise can also have an impact on gastric physiology and appetite regulation, linking nausea and anorexia to gastric malfunctioning and possibly representing a compensatory, not necessarily causative, response.

While our results strongly suggest a direct effect of GDF15 in gastric muscle wall, these initial data are obtained in rodent models. Although they represent a valid model for pre‐clinical study, further research is needed to confirm these findings in human gastric samples. In fact, we cannot exclude the hypothesis that the human stomach functions differently from that of rodents, which, for example, do not vomit.

In conclusion, the thorough identification of the mechanism of action of GDF15 is desirable to open the doors to translational perspective: elucidating if and how elevated plasma levels of this peptide can have direct repercussions in gastric physiology and appetite regulation promises possible clinical applications in the treatment of eating disorders and obesity, as well as nausea and vomiting. In this regard, many drugs used to inhibit vomiting seem to have not a scientific rationale, making novel approaches necessary for counteracting nausea, especially pointing to modulation of vagal or gastric pacemaker activity, to the usage of receptor agonists or new targets exactly like GDF15 [63, 65].

## Author Contributions

**Rachele Garella:** data curation (equal), formal analysis (equal), investigation (equal), methodology (equal), writing – original draft (equal), writing – review and editing (equal). **Francesco Palmieri:** formal analysis (equal), investigation (equal), methodology (equal), software (equal), visualization (equal), writing – review and editing (equal). **Livio Tarchi:** data curation (equal), resources (equal), writing – original draft (equal), writing – review and editing (equal). **Alessia Tani:** formal analysis (equal), investigation (equal), methodology (equal), writing – review and editing (equal). **Flaminia Chellini:** formal analysis (equal), funding acquisition (equal), investigation (equal), writing – review and editing (equal). **Giulia Guarnieri:** formal analysis (equal), methodology (equal), writing – review and editing (equal). **Annamaria Morelli:** data curation (equal), formal analysis (equal), resources (equal), writing – review and editing (equal). **Caterina Bernacchioni:** investigation (equal), methodology (equal), writing – review and editing (equal). **Paolo Rovero:** conceptualization (equal), writing – review and editing (equal). **Valdo Ricca:** conceptualization (equal), writing – review and editing (equal). **Giovanni Castellini:** conceptualization (equal), supervision (equal), writing – review and editing (equal). **Chiara Sassoli:** conceptualization (equal), data curation (equal), formal analysis (equal), funding acquisition (equal), investigation (equal), project administration (equal), resources (equal), supervision (equal), validation (equal), writing – original draft (equal), writing – review and editing (equal). **Roberta Squecco:** conceptualization (lead), data curation (equal), formal analysis (equal), funding acquisition (equal), investigation (equal), project administration (lead), resources (equal), supervision (equal), validation (equal), writing – original draft (equal), writing – review and editing (equal).

## Conflicts of Interest

The authors declare no conflicts of interest.

## Data Availability

The data that support the findings of this study are available upon reasonable request from the corresponding author.
